# P395: The Swiss standard for air quality: does micorobiological air sampling makes sense in central sterilization services (CSS)

**DOI:** 10.1186/2047-2994-2-S1-P395

**Published:** 2013-06-20

**Authors:** M Dangel, AF Widmer

**Affiliations:** 1 Division of Infectious Diseases & Hospital Epidemiology, University Hospital Basel, Basel, Switzerland

## Introduction

Routine measuring air quality by air sampling has been questioned since there is a poor association between air quality and nosocomial infections. In Switzerland, the standard called “Good practice for the preparation of sterile medical devices-2005”defines air quality standards determined by particle Class 8 (Norm EN ISO 14644-1) in idle state (nobody is in the room) and by microbiological test in operation (upper limit. 200 cfu/m^3^) to detect malfunction of air conditioning. The objective of this study was to investigate qualitatively and quantitatively the microbiological loads before and during work session in CSS of the University Hospital Basel.

## Methods

Air sampling was performed with the Microbial Air Sampler MAS-100^®^. A total of 160 litres air was aspirated during 95 seconds on Columbia-Agar and Sabouraud plates. Agars were incubated during 3 days at 35°C followed by 5% CO_2_ for 8 days at room temperature. Sabouraud plates were incubated for 21 days to 28°C to identify slow-growing moulds. Samples were taken at 6 o’clock in the morning (idle), and during the day with maximum occupancy of CSS staff (in operation).

## Results

Between 7/2008 -2/2013, a total of 209 samples were taken, 116 sample during working hours work and 93 in the absence of any staff. The Swissmedic limit of 200 cfu/m^3^ was fulfilled in 168 (80.4%) sample, respectively 75 (64.7%) during work and 93 (100%) in idle state (p<0.001).

Data presented (mean cfu/m^3^ in idle state vs in work/ *p* value).

Overall (38.9 vs 167.6/0.0001); Coagulase-negative staphylococci (21.5 vs 120.0/0.0001); *Corynebacterium* (4.0 vs 28.0/0.037); *Aspergillus ssp* (0.9 vs 2.5/0.030); *Staphylococcus aureus* (0.0 vs 0.5/0.003); Non-fermenter (0.4 vs 2.6/0.001); Gram Negative Bacilli (0.1 vs 2.1/0.001); Other (e.g. *Cladosporium ssp*, *Paecilomyces*, Gram Positive Bacilli) (11.8 vs 11.59/0.406).

## Conclusion

Microbiological sampling in CSS during working hours commonly detects pathogenic bacteria and higher counts of moulds challenging air conditioners much more than measurements in idle state. Routine sampling in the idle state should be abandoned. However, the in-operations measurements should be better defined since the number of staff likely influences the results. Our results indicate that routine measurement in operation should be performed as outlined in the standard.

## Disclosure of interest

None declared.

